# Supplementary material for: Serotonin Signalling in Flatworms: An Immunocytochemical Localisation of 5-HT7 Type of Serotonin Receptors in Opisthorchis felineus and Hymenolepis diminuta
Source: Biomolecules. 2021 Aug 15;11(8):1212. doi: 10.3390/biom11081212 (PMC8394519; doi:10.3390/biom11081212)
Supplement: Supplementary file 1 [file biomolecules-11-01212-s001.zip › biomolecules-1248487-supplementary.pdf]

Supplementary Material

```
Opisthorchis_viverrini      -------------------------------------------------------MLRGE      5
Homo_sapiens                MMDVNSSGRPDLYGHLRSFLLP--------EVGRGLPDLSPDGGADPVAGSWAPHLLS--      50
Mus_musculus                MMDVNSSGRPDLYGHLRSLILP--------EVGRRLQDLSPDGGAHSVVSSWMPHLLSG-      51
Hymenolepis_diminuta        -----------------------------------MAS--QQNDP-LTFKFW------GE      16
Hymenolepis_microstoma      -----------------------------------MAS--QQNDP-LTFKSW------GE      16
Echinococcus_granulosus     -----------------------------------MAS--QLNDPSITFKAWGW----SE      19
Mesocestoides_corti         -----------------------------------MAT--QLNDPSITFKAWGW----SE      19
Taenia_asiatica             ------------------------------------------------------------      0
Dugesia_japonica            -----------------------------------------------------MSTINST      7
Schmidtea_mediterranea      -----------------------------------------------------MSIYNSS      7
Clonorchis_sinensis         ---------------MISAAIIILFQSIVSLGADKLITVISDGEPDQFIRSHPI----DR      41
Fasciola_hepatica           ------------------------------MTDKDIISVVSTHFSYQFPSNWTLSMFDP-      29
Schistosoma_mansoni         ----------------------------------------------------------MT      2
Schistosoma_japonicum       -----MKYIPLSYKHSFKSLLIYLF-----FVYNTLLTIVN----ALNVPDWIVEERDLT      46


Opisthorchis_viverrini      AF-------------------DQFEAAKRSSNGTSEQLSQIFKGLCFSLIIFAALFGNAL      46
Homo_sapiens                ------EVTASPAPTWDAPPDNASGCG-E--QINYGRVEKVVIGSILTLITLLTIAGNCL      101
Mus_musculus                ----FPEVTASPAPTWDAPPDNVSGCG-E--QINYGRVEKVVIGSILTLITLLTIAGNCL      104
Hymenolepis_diminuta        NCPSLWGLLANGSIT-----QDQFLSSCK--PQVDIGVGKVILTVLLSILLLGTAGGNVL      69
Hymenolepis_microstoma      NCTSLWESLANGSIT-----KDQFLGSCK--PQVDIGVGKVILTILLSILLLGTAGGNIL      69
Echinococcus_granulosus     NCTLLLDLLINRSIP-----EEAFLAACK--PHAEIDVKSIILAIVLGLLLLGTAGGNML      72
Mesocestoides_corti         NCTLLLDQLVNRTIS-----EAFFLEACK--PHADIDAGTVVLAILLTLLLLGTAGGNIL      72
Taenia_asiatica             ---------------MDANPSTTVLAATQ--ILSAAAPGSIPLAVTFCIITALTILGNTL      43
Dugesia_japonica            NCTFYFQLASKNN-S--AVQVP-FYCIKF--SF--EF--EVFLIIVLTIFLVGTAGGNLL      57
Schmidtea_mediterranea      NCTLYLQLASNSNIN--ALQVP-FYCFKF--SF--DF--EMFLIVIFTVFVVGTAGGNLL      58
Clonorchis_sinensis         MRRLYA-TENHHNIG----HGN-ITHDMT--AYSPAI--SAILSIILPSIAVATIGGNFL      91
Fasciola_hepatica           --RPFT----SSLISMAVIPEP-VVCISD--SYTPVV--SGVIGFILVLIAIGTAGGNFL      78
Schistosoma_mansoni         ISQLDN-SSIKSTLS-MNKSNT-SECISE--NDSVLI--TGIIFILSLIVAIATAGGNFL      55
Schistosoma_japonicum       APQLTI-SNNESILS-VNNSSN-IECISE--NTSASV--TGIIFILSLIAAIATAGGNFL      99
                                                                                 :  ** *

Opisthorchis_viverrini      VFIAVQRFERLRRVKTNIFLVSLAMADFLVALVVMPFSACMSLMHGRWIFGNWFCDVFNA      106
Homo_sapiens                VVISVCFVKKLR-QPSNYLIVSLALADLSVAVAVMPFVSVTDLIGGKWIFGHFFCNVFIA      160
Mus_musculus                VVISVCFVKKLR-QPSNYLIVSLALADLSVAVAVMPFVSVTDLIGGKWIFGHFFCNVFIA      163
Hymenolepis_diminuta        VIIAILIVKKLR-SPTNLLIVNLAVTDFLVSILVLPFAIAY-QIFEYWPFKQAICDLYSI      127
Hymenolepis_microstoma      VIIAILIVKKLR-SPTNLLIVSLAVTDFMVSILVLPFAIAY-QIFEYWPFQQAICDLYSI      127
Echinococcus_granulosus     VIIAILIVKKLR-SPTNLLIVNLAVTDFLVSILVLPFAIAY-QILGYWPFNQIICDLYSL      130
Mesocestoides_corti         VIIAILIVKKLR-SPTNLLIVNLAVTDFLVSILVLPFAIAY-QILGYWPFNQIICNLYNI      130
Taenia_asiatica             VISAVLLVHKLR-CPSNFLIVSLAASDLLVSTTVMPFSIYL-EYRQYWGLGEVACDIFIV      101
Dugesia_japonica            VISSVAIVKKLQ-TSSNFLIVNLACSDFLVSILVLPGAVHQVIYRGQWPFSEILCDIFIS      116
Schmidtea_mediterranea      VIMSVAIVKKLQ-TPSNFLIVNLACSDFLVSILVLPGAIHQTINRSRWPFSEILCDVFIS      117
Clonorchis_sinensis         VILAVTLVKKLQ-TPSNILIVSLAFSDFFVGLLVLPFAILD-LLKGYWPFNEPLCDMYIS      149
Fasciola_hepatica           VILAVALVKKLQ-TPSNILIVSLAFSDFLVGILVLPFSIIE-ALKGYWPFNEHLCDMYIS      136
Schistosoma_mansoni         VILAVILVKKLQ-TPSNILIGSLAFSDFFVALLVLPFTIID-AYQGYWPFNEGLCDMYIS      113
Schistosoma_japonicum       VILAVILVKKLQ-TPSNILIGSLAFSDFFVALLVLPFTIID-AYQGYWPFNEGLCDMYIS      157
                            *. ::  ..:*:   :* :: .** :*: *.  *:*          * : .  *:::

Opisthorchis_viverrini      NDVLFSTASILHLCCISMERYIAIINPLSYDRKMTKSRVTLMITTTWILSILISYLPIMS      166
Homo_sapiens                MDVMCCTASIMTLCVISIDRYLGITRPLTYPVRQNGKCMAKMILSVWLLSASITLPPLFG      220
Mus_musculus                MDVMCCTASIMTLCVISIDRYLGITRPLTYPVRQNGKCMAKMILSVWLLSASITLPPLFG      223
Hymenolepis_diminuta        SDVLLCTLSILSLCTISIDRYMAITKPFQYAPKRTPKRMFIMILISWLLSAAISVSPIFG      187
Hymenolepis_microstoma      SDVLLCTLSILSLCTISIDRYMAITKPFKYAPKRTPKRMFIMILVSWLLSAAISISPIFG      187
Echinococcus_granulosus     SDVLLCTLSILSLCTISIDRYLAITKPLQYAAKRTPKRMLIMIIISWLLSAAISIPPVFG      190
Mesocestoides_corti         SDVLLCTLSILSLCTISIDRYLAITKPLQYAAKRTPKRMLLMICISWLLSAAISIPPVFG      190
Taenia_asiatica             FDVLLCTASILNLCAISIDRYLAVTKPFEYVVKRTPKRMLIMIFTAWSISALISIPPTFG      161
Dugesia_japonica            FDVILCTSSILNLCAISIDRYLVITRPLQYAVNRTPARIGTMVAISWVTSALISIPPMFG      176
Schmidtea_mediterranea      FDVILCTSSILNLCAISIDRYLVITRPLQYAVNRTPARISLMIAISWIASALISIPPLFG      177
Clonorchis_sinensis         FDVLLCTASILNLCAISIDRYLVITRPLTYVSKRTPCRMAQMIAATWIISALICIPPNIG      209
Fasciola_hepatica           FDVLLCTASILNLCAISIDRYLVITRPFTYVSRRTPCRMAEMIAAAWIISALISIPPNIG      196
Schistosoma_mansoni         FDVLLCTASILNLCAISIDRYLVITKPLTYASRRTPQRMAAMIATAWIGSALISIPPNFG      173
Schistosoma_japonicum       FDVLLCTASILNLCAISIDRYLVITKPLTYASRRTPCRMATMIAAAWIGSALISIPPNFG      217
                             **: .* **: ** **::**: : .*: *  . .   :  *:   *  *  *   * :.


Opisthorchis_viverrini      GIYTTAEYLAQRGSQHEVCEFVVNPIYAVVSSSTSFTIPSIVMVAVYVRIYIEARRQERK      226
Homo_sapiens                W--------AQNVNDDKVCLISQDFGYTIYSTAVAFYIPMSVMLFMYYQIYKAARKSAAK      272
Mus_musculus                W--------AQNVNDDKVCLISQDFGYTIYSTAVAFYIPMSVMLFMYYQIYKAARKSAAK      275
Hymenolepis_diminuta        W--------EQQN-TPFFCNYNMDLTFQIYATLTAFYIPLALMLVLYGKILVLAKQIALV      238
Hymenolepis_microstoma      W--------EQQN-APFFCNYNMDLTFQIYATLTAFYIPLTVMLVLYGKILVLAKQMAIA      238
Echinococcus_granulosus     W--------EQKN-SPFYCGYSEELTYQIYATMTAFYIPLTVMLVLYGKILVLAKQMASV      241
Mesocestoides_corti         W--------EQKN-SPFYCGYSEELTYQIYATMTAFYIPLTVMVILYGKILVLAKQMALT      241
Taenia_asiatica             F--------KDEF-VPGKCSYSQNFIYQIYACFGAFYIPLIVMLILYGRIVFLARRIVRS      212
Dugesia_japonica            L--------KETF-IPGQCNYSDNLIYQIYATFGAFYIPLIVMLILYGRIFKLAREMAQN      227
Schmidtea_mediterranea      W--------KETF-IPGQCSYSENLLYQIYATFGAFYIPLLVMLILYGRIFKLARVLAQN      228
Clonorchis_sinensis         W--------KSPF-QEGRCEYSEDVGYQIYATFCAFYLPLLVMLVLYGKIFKLAREMSRN      260
Fasciola_hepatica           W--------KTPF-EICKCEYSKNVGYQIYATFCAFYLPLLVMIVLYGRIFKLAREMSRA      247
Schistosoma_mansoni         W--------KDPF-QKCACEYSKNVGYQVYATFFAFYLPLIVMIILYGRIFKLAREMSRS      224
Schistosoma_japonicum       W--------KEPF-QKCACEYSKNVGYQVYATFFAFYLPLIVMITLYGRIFKLAREMSRS      268
                                            *    :  : : :   :* :*  :*: :* :*   *:

Opisthorchis_viverrini      IHHLHLSKKLKTSSDATTYTTE-----Q--------------------------------      249
Homo_sapiens                HKFPGFP---RVEPDS--------------------------------------------      285
Mus_musculus                HKFSGFP---RVQPES--------------------------------------------      288
Hymenolepis_diminuta        DAQTTRKGSIDTQMRD-SSIPE--------GTDCNRSSLYPPGDNC--------------      275
Hymenolepis_microstoma      DAQIARKSSTDTQMRN-SSLPE--------STDWNRSSIYPPGNNC--------------      275
Echinococcus_granulosus     DAQVGRKGSVDTQTRS-SSIP-----------EWNRSSLYAAGESY--------------      275
Mesocestoides_corti         NAQVARESSTEMQART-SSIPA--------YADWNRNSLYPTRESY--------------      278
Taenia_asiatica             DRVRLPPTPGDEKRNSIQNYAESQETDEGTSEDKTKSTTLCFGKICCHYLQKRASLDSTD      272
Dugesia_japonica            DAKLKIGISPNSSD----------------KE-----QH---------------------      245
Schmidtea_mediterranea      DAKMKIGISPNSSD----------------RE-----HQ---------------------      246
Clonorchis_sinensis         EQRQMMPCTQNNLQHQTSQTADDPNNL----N-----SQTCL------------------      293
Fasciola_hepatica           EQRQMIASSGEEVPPGTVTLKEDTIPTTPKAD-----HITLY--------QSSAP-----      289
Schistosoma_mansoni         GQSKMTAGIS--------------------------------------------------      234
Schistosoma_japonicum       GQSKVTPSTIRKSTGI----SENVSNNSPVLE-----EK--L--------QRDLQ-----      304


Opisthorchis_viverrini      --------------------PRGS---VEKET-----------------------TVPE      262
Homo_sapiens                -----------------------V------------------------------------      286
Mus_musculus                -----------------------V------------------------------------      289
Hymenolepis_diminuta        -------------------ANSQFFNDLKDNW-------DTTAGDAKSRQSVVSFKPSPV      309
Hymenolepis_microstoma      -------------------ANSQLFNDLKDNW-------EGAERGAKSRQSVVSFRPSPV      309
Echinococcus_granulosus     -------------------KASALYPDLKEEH-------KGNRGDSKPRQSVVIFKPTPV      309
Mesocestoides_corti         -------------------TSQKVYTEANDNWQQ---ILEPNNTDFKSRQSVVSFKSPPA      316
Taenia_asiatica             KPVDPNPYINSSLGHSNPTTPMVFISGANDSPRLNPPLIKGNNCPTVPIHHFSRL-PPPK      331
Dugesia_japonica            --------------------SHLR---IVDSHICNTP-----------------------      259
Schmidtea_mediterranea      --------------------PNLR---IVDSHLCSTP-----------------------      260
Clonorchis_sinensis         --------------------PRQV---ESPS----------EKCDND------------A      308
Fasciola_hepatica           --FQDNELLAT-----ESTQRRQV---MDDTHTFNNNTTENNNNNNS------------S      327
Schistosoma_mansoni         ----------------------------HDEHIVTKAIN---------------------      245
Schistosoma_japonicum       --ITDTN----------IA-EFVQ---LNDEHVVTIETS---------------------      327


Opisthorchis_viverrini      NLTNLLMRASETNHQPITN-----------------------------------------      281
Homo_sapiens                I---------ALNGIVKLQ------------------------------KEVE------      300
Mus_musculus                I---------SLNGVVKLQ------------------------------KEVE------      303
Hymenolepis_diminuta        IM--------RTNREPIKR----------RKVYGMTMFTGRSQSEILNNGRSSEYG----      347
Hymenolepis_microstoma      VM--------RTNRDPVKS----------RKVNSMMAFTRRSQSDVVNNGRSSEYS----      347
Echinococcus_granulosus     VM--------RMNRRP-YR----------GRK------INSSPSALGNNGRSLEVS----      340
Mesocestoides_corti         IR--------PPGRCQDVR----------GKFCCLFASSHRASTEITNNGKSLEQT----      354
Taenia_asiatica             SCPQSPPKENPNSDCESLSPGVGFLTPSPRRPHGLFTKQHR---VSLHPGTGNPKHRSPL      388
Dugesia_japonica            ------------NGQ--MN----L---------SLY-----------SG----------      270
Schmidtea_mediterranea      ------------NGQ--MN----L---------SLY-----------SG----------      271
Clonorchis_sinensis         FVNSVP--SGPIPGP--VD------T---TGPHPLT-----------NGKSEEDR----      339
Fasciola_hepatica           FCPNIIRETNEINGN--TSSGQNF-VINPPVTPELS-----------NGHSYAKS----      368
Schistosoma_mansoni         ------------NGV--KKDEDT------NDICKAR--------------E--YD----      264
Schistosoma_japonicum       ------------NGI--KNEGNI------KAIPELK--------------E--KD----      346
```

```
Opisthorchis_viverrini      ---------------RMSPPS--------IPNQSLESRE-----HLKIHRENKAAKTLGI   313
Homo_sapiens                ---------------------------ECANLSRLLKHERKNI--SIFKREQKAATTLGI   331
Mus_musculus                ---------------------------ECANLSRLLKHERKNI--SIFKREQKAATTLGI   334
Hymenolepis_diminuta        ----------GIISVRSMSS-----Y-GKSPITLLRRGKRRSPSDQGKGETHKAVTTLGV   391
Hymenolepis_microstoma      ----------GSVSDRNARN-----I-RKKSFQD--EGEDEDESEEDQMSKRDYETSVEI   389
Echinococcus_granulosus     ----------GS--SRSLYA-----Y-AKSPLSMLRHHRRRKPSDQGKGETHKAVTTLGV   382
Mesocestoides_corti         ---------------ASLSG-----P-TRPPLSLLRRKRRRSPSDQGKGETHKAVTTLGV   393
Taenia_asiatica             LRERAYSVTVAHLQTKNPRSSLPFFFKQRLSIALHLKRPG-----IKRSNEAKAIRTLGV   443
Dugesia_japonica            --SLCSISKSNNLSD-SS-----------PNKVNLRNRVK-----LKSSTETKAITTLGV   311
Schmidtea_mediterranea      --SLVSIIKSTNFSE-SS-----------QNKLNNRSRAK-----LKSSTETKAITTLGV   312
Clonorchis_sinensis         --IRRQTANTVTFSEDESRTRTS-F--WKPPSNPDLVRRR-----SKNSSETKVIKTLGI   389
Fasciola_hepatica           --KRCSKDSSTAFLDRSS-GTVA-G--NRQSSSVDLQRRK-----SRGSADSKVIKTLGV   417
Schistosoma_mansoni         --KRLNSYSSRKLLTDSM------N--VTSELSREAHGRR-----SRGNSDTKVIKTLGV   309
Schistosoma_japonicum       --KRLNSYSSRKLLTDSM------N--ANNELSRDAPGRR-----SRSNSDTKVIRTLGV   391
                                                                                .   :: :

Opisthorchis_viverrini      IIG-----------------------------AFILCWCPFFFWYISTNVCRAFA---DC   341
Homo_sapiens                IVG-----------------------------AFTVCWLPFFLLSTARPFICGT---SCS   359
Mus_musculus                IVG-----------------------------AFTVCWLPFFLLSTARPFICGT---SCS   362
Hymenolepis_diminuta        IMG-----------------------------CFTICWLPFFVSQTLILVLVLTGTN-QL   421
Hymenolepis_microstoma      TPGHTVFPDKGKQFETETKIFPPDSSGKLQDQQPVLCYIPGMAFPGY-------------   436
Echinococcus_granulosus     IMG-----------------------------CFTICWLPFFVSQLITPIINCFTEQ-RF   412
Mesocestoides_corti         IMG-----------------------------CFTVCWLPFFVSQLITPIINCFTKQ-KF   423
Taenia_asiatica             IMG-----------------------------VFCICWLPFFIVALGRPLYNYIHNT-EK   473
Dugesia_japonica            IMG-----------------------------CFTICWLPFFLIQISKPVLKVANVDSMN   342
Schmidtea_mediterranea      IMG-----------------------------CFTICWLPFFIIQIAKPILKVASVNSVN   343
Clonorchis_sinensis         IMG-----------------------------CFCLCWLPFFMVQLLLALLKAGKVKTDG   420
Fasciola_hepatica           IMG-----------------------------CFCLCWLPFFIVQLLLALLTASGYKTEN   448
Schistosoma_mansoni         IMG-----------------------------CFCLCWLPFFMIQLLLALLSAAGYNTVN   340
Schistosoma_japonicum       IMG-----------------------------CFCLCWLPFFMTQLLLALLSAAGYNTTN   422
                              *                                :*: * :

Opisthorchis_viverrini      PYPSILGEVLFWIGYFNSTLNPIIYAFYNRSFRRSFKHILQCHVTVKRAAQAKIDRTHSP   401
Homo_sapiens                CIPLWVERTFLWLGYANSLINPFIYAFFNRDLRTTYRSLLQCQYRNINRKLSAAGMHEAL   419
Mus_musculus                CIPLWVERTCLWLGYANSLINPFIYAFFNRDLRTTYRSLLQCQYRNINRKLSAAGMHEAL   422
Hymenolepis_diminuta        I--------------CKDRLKIAIR----LKLAHTTGFPLRF-----------TQRFVQV   452
Hymenolepis_microstoma      ------------------------------QL--PYPYPYPI-----------PQGYPNF   453
Echinococcus_granulosus     SIPPTLFQVFIWLGYGNSFLNPLLYALFNREFRLPFLYILRLQCNDINTRLRTETFSHQF   472
Mesocestoides_corti         SIPPTLFQVFVWLGYCNSFLNPLIYALFNREFRLPFIYILRFQCYSINTRLRTETFSHQF   483
Taenia_asiatica             DIDPRLSCFFLWLGYVNSALNPLIYAIFNREFRRPFWELMTCHCLSINARLRERRYQHEY   533
Dugesia_japonica            YFPIWLIELCLWLGYFNSFLNPIIYAKFNREFRTPFKQILLCKCNSINARLRSETFVQEY   402
Schmidtea_mediterranea      YFPIWLIEICLWLGYFNSFLNPIIYAKFNREFRTPFKQILLCRCSSINARLRSETFVQEY   403
Clonorchis_sinensis         LVPKACFQFLQWLGYINSSLNPLIYAKFNQEFRLPFKLILLCHCRNINARLRSAAFSAQY   480
Fasciola_hepatica           MIPVEAFRFLLWLGYVNSCLNPVIYAKYNQEFRLPFKMILLCRFRRINARLRSATFSAEY   508
Schistosoma_mansoni         MIPVSVFRFLQWLGYVNSFLNPLIYAKFDREFRGPFKMILLCHCRNINARLRAVHYSAQY   400
Schistosoma_japonicum       IIPVSVFRFLQWLGYVNSFLNPLIYAKFDREFRGPFKMILLCHCRNINARLRAAHYSAQY   482
                                                          .:

Opisthorchis_viverrini      SLRRSAQAGYLTSMQPMNRSDST---E---------------------------------   425
Homo_sapiens                KLAERPERP---EFVLQNADYCR-----KKGHDS--------------------------   445
Mus_musculus                KLAERPERS---EFVLQNCDHCG-----KKGHDT--------------------------   448
Hymenolepis_diminuta        D----DEKSYIHPITG--------------------------------------------   464
Hymenolepis_microstoma      GKKKKSGTSYTNSITS----ISSGKRRPR--------------QKSRGLSHCNVNSPSIA   495
Echinococcus_granulosus     GLPQKSRVGYSNSLSRGSSSVSGSKRQRR--------------QKPRGLSPLTNSQVQAS   518
Mesocestoides_corti         DLPRKSGLSYRNSLARGSSSSCN-RRQQR--------------RKLRGLSPLANVSPHAT   528
Taenia_asiatica             RPSPISLPTVSGGSGG-------------------VGETLSLP-RSRNSAHLLDRRHSSM   573
Dugesia_japonica            GLPNSSAPTKRGSFDQTSLNNGSFTKNRR----------RRTPGVLRQNPHLNNCK-IEN   451
Schmidtea_mediterranea      GLPNSLVNNKRGSMDQTSLNNGSFTKNRR----------RRKPGLLRQNPNLNKLNNSSD   453
Clonorchis_sinensis         GLSSSGSR--RSNSVLLSVSTRADRSSRI-----DSG-----RRRPPSQPHIVHSGASTF   528
Fasciola_hepatica           GLPSASTK--SNSWFQSISTRGT--RRNHRGSRTKRG--YDHQGRPRPLSHLHQTDTLKM   562
Schistosoma_mansoni         GLPSSSSK--RQSIVISSPYTRNDTASRWLGKSLNQRCTSAVPIRPRRRPQMNFRNAISG   458
Schistosoma_japonicum       GLPSSSNK--RQSIVASSLYSRSDLASKWFGRSLNQGCTSTLPNRPRPRPQLNFRNTVST   540

Opisthorchis_viverrini      ------------------------------------------------------------   425
Homo_sapiens                ------------------------------------------------------------   445
Mus_musculus                ------------------------------------------------------------   448
Hymenolepis_diminuta        ------------------------------------------------------------   464
Hymenolepis_microstoma      -----STIETPNVQ-IESSPAKSLT-KEEFKSNEDELDYQLTPALNNALTPPLSTPQHFF   548
Echinococcus_granulosus     PSNTSQVAEASNVE-IEKPPLRSSLNLNTVAPEEPK--LLLVQ-HLNNLPSPLHL----T   570
Mesocestoides_corti         INAAGHTKA---------PSSEHNVFVSHLSSEHDE--VLQTPHSLFDQPSRFSIPTSLF   577
Taenia_asiatica             L---AE------------------------------------------------------   576
Dugesia_japonica            S---RKIYECSLINKTNKFPPESSVLLNSIANGNTKP---VKK---DTDDNPFHFSDQIS   502
Schmidtea_mediterranea      S---RKIYECSLMKTNNSSLPESSMLLNTLVNGSAKV---LKP---DLDDNQFFFYDHMS   504
Clonorchis_sinensis         G---N-------------------------------------------------------   530
Fasciola_hepatica           E---NDPKPSPL------------------------------------------------   571
Schistosoma_mansoni         Q---TDER----------------------------------------------------   463
Schistosoma_japonicum       E---PDKR----------------------------------------------------   545

Opisthorchis_viverrini      ------------------------------------------------------------   425
Homo_sapiens                ------------------------------------------------------------   445
Mus_musculus                ------------------------------------------------------------   448
Hymenolepis_diminuta        ------------------------------------------------------------   464
Hymenolepis_microstoma      PVS---ITTGNDDLLENNPPRPSRLMSRRESLT--STRSYYDSYIPNLSVEYSRSPQPTS   603
Echinococcus_granulosus     PLQ-PNTPLEKGDLLKNNHPPPSWMMSRRESLA--SIRSNYDTYIPNLNAEFQRSPLPTP   627
Mesocestoides_corti         TASEVLANPSGNDLLENTLRICPLPNCSQESDVGIRSDRIYDTHIPYLNVQFQRRPRSSP   637
Taenia_asiatica             ------------------------------------------------------------   576
Dugesia_japonica            PN-------------------C--------SINDQRSEKMFMILVQHLNL----------   525
Schmidtea_mediterranea      PN-------------------S--------SINDQRSEKYVYDFGPASESVSIDPPPP--   535
Clonorchis_sinensis         ------------------------------------------------------------   530
Fasciola_hepatica           ------------------------------------------------------------   571
Schistosoma_mansoni         ------------------------------------------------------------   463
Schistosoma_japonicum       ------------------------------------------------------------   545

Opisthorchis_viverrini      ------------------------------------------------------------   425
Homo_sapiens                ------------------------------------------------------------   445
Mus_musculus                ------------------------------------------------------------   448
Hymenolepis_diminuta        ------------------------------------------------------------   464
Hymenolepis_microstoma      TSDPT-------NLRTWTKARDFTPTQIPPERFRRCSGMLNG--VKSDY--STEQVSLL-   651
Echinococcus_granulosus     SSAPAYLTPSMNNVKMWTKAREFRPNQVPSERFHRWCENISCTGVKSNSSLSVANP----   683
Mesocestoides_corti         SSAPPNLTTFENSLHTWTGVSDYQPKSQTCERRRRQSDAAVSPLMRNK---FQKKPRLIE   694
Taenia_asiatica             ------------------------------------------------------------   576
Dugesia_japonica            ------------------------------------------------------------   525
Schmidtea_mediterranea      ----I-------NVSSYNLNVDY-------------------------------------   547
Clonorchis_sinensis         ------------------------------------------------------------   530
Fasciola_hepatica           ------------------------------------------------------------   571
Schistosoma_mansoni         ------------------------------------------------------------   463
Schistosoma_japonicum       ------------------------------------------------------------   545

Opisthorchis_viverrini      -- 425
Homo_sapiens                -- 445
Mus_musculus                -- 448
Hymenolepis_diminuta        -- 464
Hymenolepis_microstoma      -- 651
Echinococcus_granulosus     -- 683
Mesocestoides_corti         WE 696
Taenia_asiatica             -- 576
Dugesia_japonica            -- 525
Schmidtea_mediterranea      -- 547
Clonorchis_sinensis         -- 530
Fasciola_hepatica           -- 571
Schistosoma_mansoni         -- 463
Schistosoma_japonicum       -- 545
```

Figure S1. Multiple sequence alignment of amino acid sequences of serotonin 5-HT7 receptors in cestodes, trematodes and planarian species with the annotated genome sequences.
